# Increasing connectivity enhances habitat specialists but simplifies plant–insect food webs

**DOI:** 10.1007/s00442-020-04830-6

**Published:** 2020-12-24

**Authors:** Péter Batáry, Verena Rösch, Carsten F. Dormann, Teja Tscharntke

**Affiliations:** 1 “Lendület” Landscape and Conservation Ecology, Institute of Ecology and Botany, Centre for Ecological Research, Alkotmány u. 2-4, 2163 Vácrátót, Hungary; 2 Ecosystem Analysis, Institute for Environmental Sciences, University of Koblenz-Landau, Fortstr. 7, 76829 Landau, Germany; 3 Biometry and Environmental System Analysis, University of Freiburg, Tennenbacher Str. 4, 79106 Freiburg, Germany; 4 Agroecology, University of Goettingen, Grisebachstr. 6, 37077 Göttingen, Germany

**Keywords:** Calcareous grasslands, Habitat fragmentation, Isolation, Landscape composition, Leafhoppers

## Abstract

Strong declines of grassland species diversity in small and isolated grassland patches have been observed at local and landscape scales. Here, we study how plant–herbivore interaction webs and habitat specialisation of leafhopper communities change with the size of calcareous grassland fragments and landscape connectivity. We surveyed leafhoppers and plants on 14 small (0.1–0.6 ha) and 14 large (1.2–8.8 ha) semi-natural calcareous grassland fragments in Central Germany, differing in isolation from other calcareous grasslands and in the percentage of arable land in the surrounding landscape (from simple to complex landscapes). We quantified weighted trophic links between plants and their phytophagous leafhoppers for each grassland fragment. We found that large and well-connected grassland fragments harboured a high portion of specialist leafhopper species, which in turn yielded low interaction diversity and simple plant-leafhopper food webs. In contrast, small and well-connected fragments exhibited high levels of generalism, leading to higher interaction diversity. In conclusion, food web complexity appeared to be a poor indicator for the management of insect diversity, as it is driven by specialist species, which require high connectivity of large fragments in complex landscapes. We conclude that habitat specialists should be prioritized since generalist species associated with small fragments are also widespread in the surrounding landscape matrix.

**Supplementary Information:**

The online version contains supplementary material available at 10.1007/s00442-020-04830-6.

## Introduction

Landscape-scale agricultural intensification often leads to loss, degradation and fragmentation of remaining natural habitats and poses great threats to biodiversity (Fischer and Lindenmayer 2007; Thomas 2016). Species-rich semi-natural habitats, such as most calcareous grasslands in Central and Western Europe, are threatened by such man-made landscape transformation (Poschlod and WallisDeVries 2002; Habel et al. 2013). Since these species-rich grasslands are usually embedded in an intensively managed arable matrix, both local and landscape features matter (Poniatowski et al. 2018). Another type of threat for calcareous grasslands is the complete cessation of management leading to secondary succession (Reitalu et al. 2009; Kormann et al. 2015). Losing calcareous grassland fragments to intensification (by fertilization) or abandonment (succession by shrub and tree encroachment) results in increasing isolation of the remaining fragments.

A plethora of studies have investigated effects of habitat fragmentation on species diversity, plant and animal population size, genetic diversity and ecosystem functions throughout the world (see syntheses by Aguilar et al. 2006, 2008; Magrach et al. 2014; Fontúrbel et al. 2015; Fletcher et al. 2018). Typically, habitat fragmentation has been studied at three spatial scales: the within-fragment scale (e.g. edge effects: Ries et al. 2017 or habitat quality within fragment: Poniatowski et al. 2018), the scale of the fragment itself (e.g. its area and shape: Fahrig 2003) and the landscape scale (e.g. isolation of fragments and matrix effects: Ewers and Didham 2006; Laurance et al. 2007). The landscape perspective may be particularly relevant for conservation management of species with specific traits (Hagen et al. 2012). For example, Martinson and Fagan (2014) found in a meta-analysis that habitat specialists are more negatively affected by fragmentation than habitat generalists. This is related to the reduction of dietary specialisation of herbivore communities of fragmented landscapes resulting in changes of community composition and plant–herbivore interactions of fragmented landscapes (Bagchi et al. 2018).

Understanding of habitat fragmentation effects on communities also needs to consider food web interactions (Polis et al. 1997; Miranda et al. 2013), as it explains resource-consumer relationships as well as within-guild competition. Food web research has been burgeoning in fragmentation studies of this century (Hagen et al. 2012). For example, Valladares et al. (2012) showed that habitat loss through fragmentation resulted in food web contraction around a core of highly connected species, for both plant–herbivore and host–parasitoid systems. However, the relationship between diversity and food web structure remains unclear (Rooney and McCann 2012), and particularly for antagonistic plant–herbivore food webs we have far less data than for mutualistic plant-pollinator food webs (Miranda et al. 2013). This may be due to the difficulty to observe and quantify herbivory, as often many herbivore groups are involved (but see e.g. Valladares et al. 2012; Rossetti et al. 2019). However, herbivore-plant interactions can be also estimated in a retrospective and indirect way, as shown by Woodcock et al. (2012), who created plant–herbivore beetle food webs based on literature data on herbivore food preferences and detailed surveys of plant and herbivore amounts, an approach common to food webs, but much less so to interaction networks (Dormann and Blüthgen 2017).

With decreasing fragment size, the core area of fragments becomes smaller and less suitable for habitat specialists (Didham 2010). Thus, one might expect that loss of habitat has consequences for food webs in isolated and small fragments as well since many of the habitat specialist species can be characterized by limited dispersal capacity and more specialised food consumption. This holds also for plant–herbivore food webs through bottom-up constraints of resource availability and quality as shown by Bagchi et al. (2018), who investigated the effects of fragment size and isolation jointly. However, it remains unknown, whether and how fragment size, isolation and landscape matrix do concurrently modulate food web structure through habitat speciality. Therefore, here we analyse how plant–herbivore food webs are related to the level of the herbivores’ habitat specialisation in fragmented habitats. We focus on quantitative plant–leafhopper food webs of small vs. large calcareous grassland fragments along landscape gradients of proportions of arable land (an inverse of landscape complexity) and patch isolation (or patch connectivity, respectively). Studies on the same grassland patches showed that plant richness increases with increasing connectivity of fragments and that it is negatively affected by high-intensity agriculture in the surrounding matrix (Rösch et al. 2015). We thus hypothesize that (1) leafhopper communities are dominated by generalist species in smaller and more isolated fragments embedded in a landscape matrix dominated by hostile arable land. (2) Interaction diversity is expected to increase with fragment size and connectivity, paralleling an increasing proportion of specialists.

## Materials and methods

### Study area and study design

The study area was located around the city of Göttingen in southern Lower Saxony in central Germany. It is characterised by intensively managed agricultural areas dominated by cereal, oilseed rape and maize fields and fertile meadows, interspersed with beech forests and patchily distributed fragments of calcareous grasslands (for a map see Rösch et al. 2013). The calcareous grasslands belong to the plant association *Mesobrometum erecti* Koch 1926 (Ellenberg and Leuschner 2010), and about 70% of them are smaller than one hectare (Rösch et al. 2015). They are managed by mowing or by grazing with sheep, goats, cattle or horses. To study the effects of habitat fragmentation on plant-leafhopper food webs, we selected 14 small (mean ± SEM: 0.33 ± 0.04 ha, range 0.06–0.60 ha) and 14 large (mean ± SEM: 3.70 ± 0.60 ha, range 1.24–8.76 ha) fragments of calcareous grassland in 2010, along two orthogonal gradients: isolation from other calcareous grasslands (connectivity index: 20–849 within a radius of 2000 m, Hanski et al. 2000) and composition of the surrounding landscape (arable land: 27–77% within a radius of 500 m). This was achieved after analysing digital maps (ATKIS-DLM 25/1 Landesvermessung und Geobasisinformationen Niedersachsen 1991–1996, Hannover, Germany) with the geographical information system ArcGIS 10.0 (ESRI Geoinformatik GmbH, Hannover, Germany) and subsequent extensive field surveys in the study area (for further details on site selection see Rösch et al. 2013). There was no strong correlation among the three design variables (Spearman’s correlation for fragment type vs. connectivity index ρ = − 0.20; fragment type vs. arable % ρ = 0.22; connectivity index vs. arable % ρ = − 0.17). Additionally, habitat quality characterised by food plant species richness of leafhoppers was independent of fragment size (GLM with Poisson distribution, *t*_26_ = 0.655, *p* = 0.518) in contrast to other studies (Helbing et al. 2017), and thus probably did not bias our results. Finally, some of our study fragments were grazed or mown, whereas in some management had been abandoned. Mowing was done at different times throughout the year, but never before the first insect sampling. We could not avoid differences in habitat management, but to assure that fragments exhibited characteristics of calcareous grasslands, we only included those that harboured more than ten of the plant species typical for calcareous grasslands in the study area (Krauss et al. 2004).

### Sampling methods

At the beginning of June 2010, we designated six transects within homogeneous vegetation per fragment for surveying plants and leafhoppers (Hemiptera: Auchenorrhyncha). The transects were well spread across the fragments, about 10–15 m away from each other within a fragment (minimum distance 3 m); fragment edges were avoided. We recorded the cover (%) of vascular plant species, bare ground cover (%) and litter cover (%) of each transect in botanical plots (one 1 × 5 m plot per transect). Subsequently, we calculated the mean relative cover of each species and the total number of plant species (i.e. species richness per 30 m^2^) for each fragment. Relative cover (%) per species was calculated by dividing the cover of the given species by total plant cover plus bare ground cover and litter cover.

We sampled leafhoppers by sweep netting (heavy-duty sweep net, 7215HS, diameter 38 cm; BioQuip) centred on the botanical plots (20 sweeps each, i.e. 120 sweeps in total) during dry weather on three occasions (at the beginning of June, at the end of July and at the beginning of September in 2010). These sweep-net transects exceeded the botanical plots in size and were approximately 10 m long. The specimens of leafhoppers caught were transferred into ethanol (70% vol) and subsequently identified to species level (Rösch et al. 2013). For each fragment, we pooled the leafhopper data by tallying the leafhopper species richness and summing individuals per species over the six transects and three sampling occasions.

We used the classification dataset of leafhopper species of Rösch et al. (2013), who classified species into habitat specialists and generalists according to (1) their specific habitat requirements typical for calcareous grassland (i.e. warm and dry habitat conditions, short, grazed swards, open soil) and (2) diet preferences (i.e. utilizing plants that exclusively occur on calcareous grasslands) based on Nickel and Remane (2002) and Nickel (2003). A species was classified as a habitat specialist when condition (1) or (2) were fulfilled (Prugh et al. 2008).

### Food webs

We compiled quantitative plant-leafhopper food webs for each calcareous grassland fragment based on the cover of food plant species and food preferences of leafhoppers provided in the literature (Nickel and Remane 2002; Nickel 2003). We caught 76 leafhopper species (Rösch et al. 2013). However, for compiling food webs, we excluded one leafhopper species that could not be identified to species level since only female specimens were caught, four leafhopper species as their food plants were unknown at genus or species level, and four further leafhopper species because their food plants had not been recorded on the transects in the given fragment (Online Resource 1). This resulted in a leafhopper dataset of 67 species with 6706 specimens (5% of all specimens were excluded, see Online Resource 1). Monophagous species and species with only one food plant in the fragment were assumed to feed on that plant species only. Following the approach of Woodcock et al. (2012), the abundance of species with multiple potential food plants was split proportionally to percentage cover of food plants within fragments. Finally, we quantified trophic interaction networks of food plants and leafhoppers for each fragment by using the bipartite package version 2.11 (Dormann et al. 2009) of the statistical software R (R Development Core Team 2020).

### Statistical analysis

For characterizing the 28 food webs, we calculated weighted linkage density (links per species in a network), generality (mean number of plant species per leafhopper species) and interaction diversity (Shannon diversity of interactions) (Dormann et al. 2009). For analyses of food web indices, we used linear regressions with the following explanatory variables: (1) percentage of arable land, (2) fragment size (as a factor, either ‘large’ or ‘small’), (3) connectivity (log-transformed to accommodate its non-linear effect), and (4) their two-way interactions. In the case of species richness ratio of specialist and generalist leafhoppers, we fitted binomial model (Generalized Linear Model) with the same explanatory variables. Additionally, in each model we included (5) the sum of the food web’s plant and leafhopper species richness per fragment as a control covariate since food web structure and biodiversity can be tightly linked (Rooney and McCann 2012). In this way, we assess the change in food web structure in excess to what we would expect form changes in species richness. All continuous explanatory variables were standardized from zero to one to put them on the same scale. We performed model diagnostics to test for normal distribution of model residuals by investigating normal quantile–quantile plots and plotting model residuals against fitted values to visualize error distribution and potential heteroscedasticity. We calculated all models (including null model) nested in the global model using the R package ‘MuMIn’ (Bartoń 2020), and compared them based on Akaike Information Criterion corrected for small sample size (AICc). We performed model averaging if the top model and subsequent models differed less than six units in AICc. Model-averaged parameter estimates were calculated over the subset of models including the parameter (conditional average) to avoid shrinkage towards zero. Models did not present variance inflation due to independent variables (largest VIF < 1.15, Zuur et al. 2009).

## Results

**Fig. 1:**
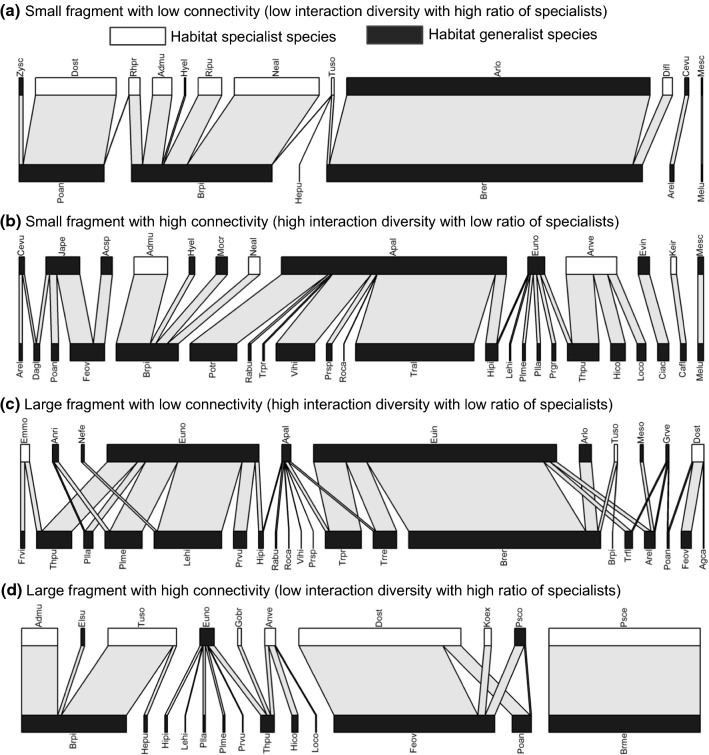
Example bipartite food webs showing trophic links between leafhoppers and food plants for **a** a small fragment with low connectivity, **b** a small fragment with high connectivity, **c** a large fragment with low connectivity and **d** a large fragment with high connectivity. Individual leafhopper species are represented by boxes on the upper level, the length of which is proportional to the abundance of that leafhopper species. Habitat specialist leafhoppers are marked with white boxes, habitat generalists with black boxes. Individual plant species are represented by boxes on the lower level, the width of these boxes is proportional to the abundance of leafhoppers with feeding associations with those plants. Species abbreviations refer to the first and second letters of the generic and specific names, which are available in the Supplementary online material (Online Resource 1)

In the 28 fragments of calcareous grassland, we recorded 167 plant species, of which 65 species represented food plants in the realized food webs (Online Resource 1). Of the 67 leafhopper species present in the food webs, 38 species with 2524 specimens were classified as habitat generalist and 29 species with 4182 specimens as habitat specialist (Online Resource 1). 24% of habitat generalist species and 66% of habitat specialist species were monophagous species. The 28 food webs contained altogether 968 plant–herbivore interactions (Fig. 1).

**Fig. 2:**
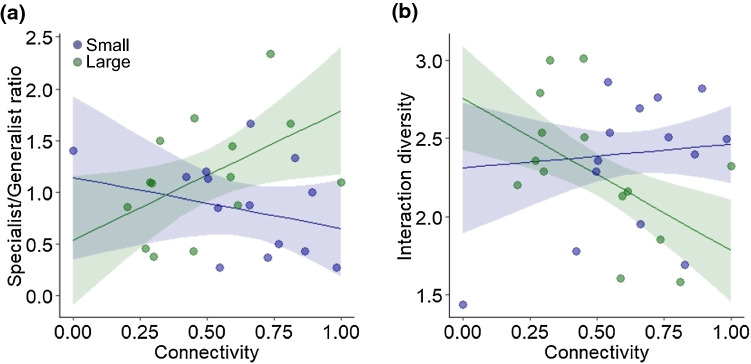
**a** Effect of patch isolation measured by a connectivity index (Hanski et al. 2000, log-transformed) on the ratio of specialist/generalist species richness of leafhoppers in conjunction with fragment size (Small: 0.1–0.6 ha shown with blue colour, Large: 1.2–8.6 ha shown with green colour). **b** Effect of patch isolation and fragment size on interaction diversity of leafhopper food webs. The lines show fitted regression lines with confidence intervals

In the analysis of the ratio of specialist and generalist leafhopper species richness, we found an interaction between fragment size and patch connectivity (Table 1). An increase in patch connectivity caused a decrease in specialist/generalist ratio in small fragments, but an increase of that ratio in large fragments (Fig. 2a). In the case of food web linkage density, we found only a positive effect of the control covariate total species richness. Food web generality was not significantly affected by the explanatory variables. Food web interaction diversity increased with patch connectivity in small fragments, but decreased in large fragments (Fig. 2b).

**Table 1:** Summary table of general linear models after multimodel averaging of best candidate models testing for the effects of landscape composition (% arable land), fragment size (small vs. large), connectivity (described by Hanski et al. 2000, log10-transformed), their two-way interactions and pooled species richness of food plants and leafhoppers per food web on the ratio of specialist/generalist species richness of leafhoppers and weighted quantitative herbivore food web properties (linkage density, generality and interaction diversity)

Model	*R*^2^	Variable	Importance	Multimodel estimate ± 95% CI
Specialist/Generalist	0.39	Species richness	9	− 0.478 ± 0.729
		Landscape composition (L)	13	0.026 ± 0.123
		Fragment size (S)	15	− 1.017 ± 2.962
		Connectivity (C)	15	0.100 ± 2.422
		L × C	5	− 0.050 ± 0.060
		S × C	8	1.134 ± 0.977*
		L × S	2	0.003 ± 0.034
Linkage density	0.42	Species richness	8	0.586 ± 0.385**
		Landscape composition (L)	3	0.001 ± 0.009
		Fragment size (S)	4	− 0.033 ± 0.739
		Connectivity (C)	4	− 0.035 ± 0.331
		S × C	1	− 0.397 ± 0.567
Generality	0.21	Species richness	7	0.515 ± 0.644
		Landscape composition (L)	6	0.001 ± 0.016
		Fragment size (S)	7	0.300 ± 1.506
		Connectivity (C)	7	0.014 ± 0.581
		S × C	1	− 0.872 ± 0.947
Interaction diversity	0.76	Species richness	15	1.093 ± 0.461***
		Landscape composition (L)	10	− 0.041 ± 0.096
		Fragment size (S)	10	0.954 ± 1.773
		Connectivity (C)	11	− 0.434 ± 2.010
		L × C	4	0.036 ± 0.037
		S × C	5	− 0.700 ± 0.558*
		L × S	2	− 0.006 ± 0.019

The table presents the multiple *R*^2^ of the full model, the relative importance of each explanatory variable and its estimate with 95% CI after multimodel averaging. Relative importance: each variable’s importance within the best candidate models (ΔAIC < 6). Significance levels: *: < 0.05, **: < 0.01, ***: < 0.001

## Discussion

In this study, we found that the connectivity of fragments moderated the ratio of habitat specialist and generalist leafhoppers as well as the interaction diversity of plant–leafhopper food webs. Increasing connectivity in small fragments decreased the ratio of specialists to generalists, leading to higher interaction diversity of their food webs, while in large fragments the ratio of specialists to generalists increased, leading to lower interaction diversity. Hence, increasing percentages of specialists resulted in more simplified food webs in the well-connected and large fragments.

Habitat specialists are generally more affected by fragmentation than habitat generalists (Ewers and Didham 2006). A bottom-up mechanism behind this might be that in small and isolated grassland patches the availability of suitable food plants can be limited constraining specialist species (Bagchi et al. 2018). For example, Öckinger et al. (2010), analysing a large set of butterfly studies, found that species with low mobility, a narrow feeding niche and low reproduction were most strongly affected by habitat loss. According to Ewers and Didham (2006), the matrix tolerance of species can also be important. Habitat specialists are expected to be more confined to the interior areas of the remaining fragments, less tolerating edge effects than habitat generalists (Hagen et al. 2012). Generalist species might not only benefit from edge areas but can exploit other resources in the neighbouring matrix needed for their life cycle (called habitat complementation: Tscharntke et al. 2012). In contrast, specialists might be retained only in grassland patches that provide their food plants and/or their narrow environmental requirements (Hagen et al. 2012). In the light of this, Poniatowski et al. (2018), also studying calcareous grassland fragments, found that primarily habitat quality and less landscape-scale variables determine patch occupancy of given specialist insect species.

In our study, however, the focus was on larger spatial scales, and within-patch habitat quality expressed as food plant richness, was independent of fragment size. Still, we acknowledge that other non-measured quality variables, such as vegetation structure or microclimate, might have also affected leafhopper communities and their food web structure. In a recent meta-analysis on fragmentation effect on herbivores, Rossetti et al. (2017) found that food plant specialists are most vulnerable to reduced area and increased isolation of remaining fragments. We found, however, not a generally negative effect of decreasing fragment size and increasing isolation on specialists, but an interaction of these two major fragmentation effects on the prevalence of specialists. In small fragments, the ratio of specialists to generalists decreased with increasing connectivity. This is in contrast to the general expectation that specialists benefit from connectivity (see Horváth et al. (2013) with an example of grassland spider communities). Generalist species seem to be able to reach and colonise these small fragments much easier if they are better connected, resulting in a higher ratio of generalists. On the contrary, in large fragments we found that specialists profit from connectivity most. This might be explained by the higher habitat quality offered by the larger fragments, in particular, the larger population size and associated lower extinction probability. They are also less threatened by temporary or complete management cessation (Rösch et al. 2013). During the last two decades, we observed a loss of smaller grassland fragments due to abandonment, which generally threatens the existence of oligotrophic grasslands (Habel et al. 2013). Similar to our results, Horváth et al. (2013) showed that habitat specialist spiders of sandy grassland fragments prefer larger and high-quality fragments. In our case, the larger fragments might provide higher amounts and better quality of food plants for specialist (Poniatowski et al. 2018), which better meet their narrower habitat requirements than that of the habitat generalists resulting in a dominance of specialists. This is why larger fragments, embedded in landscapes with high patch connectivity, may gain even specialists from the landscape matrix. Nevertheless, we could not show any effect of arable land cover in the matrix on either the ratio of specialist and generalist leafhopper species richness or any of the food web indices. However, in our former study (Rösch et al. 2013), when we analysed the species richness of generalist leafhoppers separately, we found that connectivity plays a more important role in simple than in complex landscapes, where matrix permeability is in general lower. Finally, analysing ten butterfly fragmentation studies together, Öckinger et al. (2012) also showed that matrix quality has a weaker effect in more complex landscapes, where more resources are available, so the smaller fragments suffer less from edge effect and barriers are also less expressed.

Analysing food web structure we found that linkage density was not affected by habitat fragmentation, but increased with species richness. This positive relationship between linkage density and species richness is typical of food webs (e.g. Banašek-Richter et al. 2009). Most interestingly, interaction diversity was affected by fragment size and isolation. Food web interaction diversity increased with fragment connectivity in small fragments but decreased with fragment connectivity in large fragments. Valladares et al. (2012) found that a better connectivity of small fragments may buffer the negative effects of habitat fragmentation. This also means that habitat fragmentation effects differently filter specialists, and thereby determine the diversity of their food webs in concert.

Even though our study is based on a relatively low number of site replicates, our results demonstrate that large and well-connected grassland fragments harbour a high proportion of habitat specialist species, which is also reflected in simplified, specialized food webs. In contrast, small and well-connected fragments were characterized by higher food web interaction diversity, due to some high generalist species. This shows that the interpretation of food web metrics is rather complicated and potentially misleading. If the conservation aim is to protect habitat specialist species, then we should focus on the simple food webs in large and well-connected fragments, as found in complex landscapes. If, however, the aim is to conserve the full range of biodiversity associated with calcareous grasslands and to buffer against current and future environmental change with complex food webs through high interaction diversity, then both small and large fragments with highly connective landscapes in the surrounding should be maintained (Diacon-Bolli et al. 2012; Grass et al. 2019). Nevertheless, we think that priority should be given to habitat specialists (Poniatowski et al. 2018) since many generalist species associated with small fragments are also widespread in the surrounding landscape matrix.

## Supplementary Information

Below is the link to the electronic supplementary material.

Supplementary file1 (PDF 200 KB)

## Data Availability

Raw data available as supplementary material in Table S6 of Rösch et al. 2015 Oecologia 179: 209–222.
