# Supplementary material for: Increasing connectivity enhances habitat specialists but simplifies plant–insect food webs
Source: Oecologia. 2020 Dec 24;195(2):539–46. doi: 10.1007/s00442-020-04830-6 (PMC7882472; doi:10.1007/s00442-020-04830-6)
Supplement: Supplementary file 1 — Supplementary file1 (PDF 200 KB) [file 442_2020_4830_MOESM1_ESM.pdf]

Supplementary material

Online Resource 1 Summary information on leafhopper food plants.
List of the 67 leafhopper species with their total abundances and the potential food plants, which were recorded on the 28 fragments and used for the food webs. Habitat specialist species are labelled with an asterisk. Information on food plants and habitat specialisation were derived from Nickel (2003) and Nickel & Remane (2002).

Leafhopper species	Code	Abundance	Food plants
Acanthodelphax denticauda	Acde	1	*Deschampsia cespitosa*
Acanthodelphax spinosa	Acsp	129	*Festuca ovina*
*Adarrus multinotatus**	Admu	530	*Brachypodium pinnatum*
Allygus mixtus	Almi	1	*Acer, Quercus*
Anakelisia perspicillata	Anpe	72	*Carex flacca*
*Anaceratagallia ribauti**	Anri	162	*Plantago lanceolata, Plantago media*
*Anaceratagallia venosa**	Anve	66	*Hippocrepis comosa, Lotus corniculatus, Thymus pulegioides*
Aphrophora alni	Apal	127	*Betula, Corylus, Filipendula, Hieracium, Populus, Prunus spinosa, Ranunculus, Rosa, Trifolium, Viola*
Arocephalus longiceps	Arlo	401	*Bromus erectus, Holcus lanatus*
*Arocephalus punctum**	Arpu	2	*Festuca ovina*
Arthaldeus pascuellus	Arpa	51	*Festuca, Lolium, Poa*
Athysanus argentarius	Atar	23	*Arrhenatherum elatius, Dactylis glomerata, Deschampsia cespitosa, Holcus*
*Batracomorphus irroratus**	Bair	10	*Helianthemum nummularium*
Cercopis vulnerata	Cevu	18	*Aegopodium, Arrhenatherum, Arrhenatherum elatius, Dactylis glomerata, Filipendula*
*Chlorita paolii**	Chpa	1	*Achillea millefolium*
Cicadella viridis	Civi	2	*Carex, Juncus*
Cicadula persimilis	Cipe	15	*Dactylis glomerata*
*Delphacinus mesomelas**	Deme	1	*Festuca ovina*
Deltocephalus pulicaris	Depu	1	*Agrostis, Dactylis, Lolium, Poa*
Dicranotropis hamata	Diha	4	*Dactylis glomerata, Holcus lanatus*
*Diplocolenus bohemani**	Dibo	133	*Bromus erectus*
*Ditropsis flavipes**	Difl	151	*Bromus erectus*
*Doratura stylata**	Dost	600	*Agrostis capillaris, Festuca ovina, Poa angustifolia*
Elymana sulphurella	Elsu	42	*Brachypodium pinnatum, Holcus lanatus*
*Emelyanoviana mollicula**	Emmo	77	*Fragaria, Heracleum, Origanum, Salvia pratensis, Thymus*
Errastunus ocellaris	Eroc	30	*Dactylis glomerata, Holcus*
*Eupelix cuspidata**	Eucu	13	*Festuca ovina*
Eupteryx notata	Euno	138	*Hieracium pilosella, Leontodon hispidus, Plantago, Prunella, Thymus*
Euscelis incisus	Euin	157	*Arrhenatherum elatius, Briza media, Bromus erectus, Trifolium, Trisetum flavescens*
Evacanthus interruptus	Evin	16	*Cirsium, Senecio*

Online Resource 1. Continued.

Leafhopper species	Code	Abundance	Food plants
Fieberiella septentrionalis	Fise	7	*Ligustrum vulgare, Prunus spinosa, Rosa, Rubus idaeus*
Forcipata citrinella	Foci	22	*Carex flacca*
*Goniagnathus brevis**	Gobr	7	*Thymus pulegioides*
Graphocraerus ventralis	Grve	2	*Anthoxanthum odoratum, Arrhenatherum elatius, Helictotrichon pubescens, Poa angustifolia, Trisetum flavescens*
*Hephathus nanus**	Hena	2	*Cirsium acaule*
Hesium domino	Hedo	8	*Alnus, Betula pendula, Festuca rubra, Holcus mollis, Ulmus*
Hyledelphax elegantula	Hyel	8	*Brachypodium pinnatum*
*Jassidaeus lugubris**	Jalu	1	*Festuca ovina,*
Javesella pellucida	Jape	120	*Agrostis, Dactylis, Deschampsia, Festuca, Lolium, Poa*
*Kelisia irregulata**	Keir	145	*Carex flacca*
*Kosswigianella exigua**	Koex	67	*Festuca ovina*
Megophthalmus scanicus	Mesc	8	*Medicago lupulina, Trifolium dubium,*
Megadelphax sordidula	Meso	74	*Arrhenatherum elatius*
Mocydiopsis attenuata	Moat	1	*Festuca ovina*
*Mocydia crocea**	Mocr	289	*Brachypodium pinnatum, Bromus erectus,*
*Neoaliturus fenestratus**	Nefe	3	*Leontodon*
*Neophilaenus albipennis**	Neal	247	*Brachypodium pinnatum*
Neophilaenus campestris	Neca	41	*Agrostis capillaris, Arrhenatherum elatius*
Neophilaenus lineatus	Neli	16	*Agrostis, Carex, Deschampsia, Festuca, Holcus*
Oncopsis flavicollis	Onfl	7	*Betula pendula*
Philaenus spumarius	Phsp	357	*Cirsium arvense*
*Platymetopius major**	Plma	2	*Acer, Betula, Crataegus, Fagus, Helianthemum nummularium, Prunus, Quercus*
*Psammotettix cephalotes**	Psce	245	*Briza media*
Psammotettix confinis	Psco	19	*Agrostis, Festuca, Lolium, Poa*
*Rhopalopyx adumbrata**	Rhad	41	*Festuca ovina*
*Rhopalopyx preyssleri**	Rhpr	38	*Brachypodium pinnatum, Poa angustifolia*
*Rhopalopyx vitripennis**	Rhvi	14	*Festuca ovina*
*Rhytistylus proceps**	Rhpr	6	*Festuca ovina*
*Ribautodelphax albostriata**	Rial	101	*Poa angustifolia*
*Ribautodelphax pungens**	Ripu	187	*Brachypodium pinnatum*
Speudotettix subfusculus	Spsu	1	*Populus, Prunus*
Stenocranus minutus	Stmi	38	*Dactylis glomerata*
Streptanus marginatus	Stma	47	*Festuca ovina*
Thamnotettix confinis	Thco	2	*Quercus*
*Turrutus socialis**	Tuso	1291	*Brachypodium pinnatum, Bromus erectus, Helictotrichon pubescens*

Online Resource 1. Continued.

Leafhopper species	Code	Abundance	Food plants
Verdanus abdominalis	Veab	228	*Agrostis capillaris, Holcus lanatus*
Zyginidia scutellaris	Zysc	40	*Dactylis, Festuca, Poa*

Bellow a list of excluded species with reasons for exclusion.
Species could not be identified at species level: *Aphrodes sp.*
Food plants unknown at genus or species level: *Asiraca clavicornis*, *Macrosteles laevis*, *Psammotettix alienus*, *Psammotettix helvolus*.
Food plants not recorded on survey transects: *Balclutha punctate*, *Criomorphus albomarginatus*, *Idiodonus cruentatus*, *Psammotettix kolosvarensis*.

References

Nickel, H. & Remane, R. (2002). Check list of the planthoppers and leafhoppers of Germany, with notes on food plants, diet width, life cycles, geographic range and conservation status (Hemiptera, Fulgoromorpha and Cicadomorpha). *Beiträge zur Zikadenkunde*, 5, 27–64.

Nickel, H. (2003). *The leafhoppers and planthoppers of Germany (Hemiptera, Auchenorrhyncha): patterns and strategies in a highly diverse group of phytophagous insects*. Pensoft, Sofia and Moscow.
